# Combination of Deep Recurrent Neural Networks and Conditional Random Fields for Extracting Adverse Drug Reactions from User Reviews

**DOI:** 10.1155/2017/9451342

**Published:** 2017-09-05

**Authors:** Elena Tutubalina, Sergey Nikolenko

**Affiliations:** ^1^Kazan (Volga Region) Federal University, Kazan, Russia; ^2^Steklov Institute of Mathematics, St. Petersburg, Russia

## Abstract

Adverse drug reactions (ADRs) are an essential part of the analysis of drug use, measuring drug use benefits, and making policy decisions. Traditional channels for identifying ADRs are reliable but very slow and only produce a small amount of data. Text reviews, either on specialized web sites or in general-purpose social networks, may lead to a data source of unprecedented size, but identifying ADRs in free-form text is a challenging natural language processing problem. In this work, we propose a novel model for this problem, uniting recurrent neural architectures and conditional random fields. We evaluate our model with a comprehensive experimental study, showing improvements over state-of-the-art methods of ADR extraction.

## 1. Introduction

Recent studies on text mining applications increasingly employ nonstandard sources of information to obtain new data related to health conditions, the efficiency of treatment, drug reactions, and interactions between different drugs. Users provide information about themselves through social media posts and free-text forum comments. This rich source of information has been successfully used, for instance, to monitor adverse drug reactions, making it possible to detect rare and underestimated reactions through the users complaining about their health [1].

In this work, we focus on the identification of adverse drug reactions (ADRs). ADRs are an essential part of drug postmarketing surveillance. Traditionally, reports about ADRs have been identified using (i) FDA's Adverse Event Reporting System (AERS) complaints from individual patients and their physicians and (ii) scientific literature and reports on clinical trials [2, 3]. Nowadays, drug reactions can be extracted from user reviews provided on the Web, and processing this information in an automated way represents a novel and exciting approach to personalized medicine and wide-scale drug tests. Our goal is to extract phrases about ADRs in the context of a user's post. For example, a sentence “1st pill taken with food, a few hours after I experienced shortness of breath, a sense of depression, cramping, upset stomach” contains four ADRs, namely, *shortness of breath*, *depression*, *cramping*, and *upset stomach*. Formally, this challenging task is divided into the two subtasks: identification of ADRs and normalization of ADRs. In this paper, we focus on the first subtask.

Bidirectional recurrent neural networks (RNN) and conditional random fields (CRF) are considered to be among the most powerful models for sequence modeling [4–13], each one having its own advantages and disadvantages. In a direct RNN application, especially with LSTM or GRU cells, one can get a better model for long sequences of inputs, but the RNN output (a softmax layer) will classify every tag independently. CRF can solve this problem but is less expressive than RNN in modeling the sequence itself; therefore, it is natural to try to unite the two.

In this work, we apply a combination of RNN and CRF using the following strategy. We feed word-level representations into a bidirectional RNN to encode the context vectors for each word. On top of this RNN, we use a sequential CRF to jointly decode the words' labels for the entire sentence. A similar strategy has been successfully proposed in the past for two sequence labeling tasks: part-of-speech (POS) tagging and named entity recognition (NER) [14–16]. We evaluate our model for ADR extraction on an annotated corpus CADEC. The CADEC corpus consists of 1250 medical forum posts taken from AskaPatient.com [17], where each post has been manually annotated with mentions of ADRs. Our results show that the joint model of RNN and CRF improves the performance of state-of-the-art CRF and RNN models trained separately. Hence, we can summarize the contributions of this work as follows: (i) we have introduced a joint model that combines CRF and RNN to model the sequence of labels for ADR extraction; (ii) we have conducted empirical evaluation of this model on benchmark datasets; and (iii) experimental results have shown that the proposed model improves over state-of-the-art performance.

The paper is organized as follows: in Section 2, we survey related work; Section 3 introduces the combined RNN + CRF model, and Section 4 considers in detail our experimental evaluation. We conclude in Section 5.

## 2. Related Work

Our work represents a new look at the recently popular studies on biomedical text mining and pharmacovigilance from social media.

### 2.1. Biomedical Text Mining

Recent studies in various fields of biomedical research have applied text mining, including such problems as named entity recognition [10, 11], relation extraction [18, 19], text classification [9, 20], hypothesis generation [21, 22], and the creation of knowledge sources and linguistic resources. A comprehensive review of important areas of biomedical text mining can be found in [23, 24]. Huang and Lu [23] reported a series of evaluations of natural language processing (NLP) systems for various biomedical tasks, including both knowledge-based methods and machine learning approaches to NLP.

In general, biomedical named entities include genes/proteins, chemicals, drugs, and diseases. As for relations, most research studies have focused on the entities' functions (e.g., gene functions), relational events, and interactions (e.g., drug-drug or protein-protein interactions). Many studies have employed simple classifiers to extract information from texts. For example, Ngo et al. [25] employed a classification method on a set of features based on distributed representations to predict drug-disease relations in cancer treatment. Rastegar-Mojarad et al. [26] used machine learning methods to identify disease names from user reviews for about top 180 most frequently searched medications on the *WebMD* forum. In order to identify candidates for drug repurposing, the authors removed indications of known drugs and did a manual review of the comments without using FDA reports. The main limitation of this work is the lack of an annotated corpus to evaluate the proposed system. Zhang et al. [20] proposed a weighted average ensemble of four classifiers, based respectively on a handmade lexicon, *n*-grams, and word representation vectors (also called word embeddings). Avillach et al. [3] developed a method to find previously established relationships between drugs and adverse events using the MEDLINE corpus and medical subject headings and subheadings such as “chemically induced” and “adverse effects.” Well-recognized limitations of these resources include the need of volunteers to report events and lack of sufficiently large result sets to draw the statistical conclusion. These drawbacks have led to the rise of pharmacovigilance from social media.

### 2.2. Pharmacovigilance from Social Media

Social media has been increasingly used for medical and pharmacological research since the early 2010s; the term “pharmacovigilance” was coined for automated monitoring of social media for potentially adverse drug effects and interactions.

NLP techniques have been applied in five main domain of texts: (i) biomedical literature, clinical trial records, and electronic medical/health records (e.g., medical correspondence and letters) [3, 5, 10, 27–30]; (ii) short messages from Twitter [9, 31, 32]; (iii) user reviews from health-related and e-commerce websites [4, 26, 33, 34]; (iv) web search logs [22]; and (v) forum discussions and message boards about medications, health conditions, treatment modality, and so on [35–37]. Most of these works focused on creating linguistic methods based on keywords for extracting major adverse effects, classifiers to detect whether a text contains ADRs or is relevant to drug reactions, and sequence labeling algorithms to extract mentions of ADRs. A review of techniques applied to drug reaction detection has been given in [38, 39].

In opinion mining, one of the major tasks is the identification of opinion targets (also called aspects) or opinion expressions. This task has been studied by many researchers using frequency-based methods and unsupervised and supervised methods. In [40], authors described linguistic resources for event extraction: linguistics databases and vocabularies such as thesauri. Currently, most of the state-of-the-art methods are based on CRF with a set of hand-crafted features and bidirectional RNNs [7, 8, 10]. Irsoy and Cardie [7] applied deep RNNs to extract direct or expressive subjective expressions; in their experiments, 3-layer RNN outperformed CRF, semi-CRF, and 1-layer (i.e., shallow) RNN. Liu et al. [8] applied RNNs for aspect extraction from data sets about laptops and restaurants, and RNNs based on pretrained word embeddings outperformed feature-rich CRF-based models.

In recent years, there has been a growing interest in the area of detecting ADRs from social media. It started in 2010 with a pioneering study of Leaman et al. [41], who analyzed user posts regarding six drugs from the health-related social network *DailyStrength*. FDA alerts were used as a gold standard to evaluate discovered associations between drugs and ADRs. Yang et al. [42] conducted an experiment for ten drugs and five ADRs to examine associations between them on texts from online healthcare communities using association mining techniques. Rastegar-Mojarad et al. [26] developed a rule-based system to extract drug effects. Feldman et al. [37] identified ADRs on texts from health-related online forums. They employed dictionary-based drug detection, and symptoms were extracted with a combination of dictionary-based and pattern-based methods. Pointwise mutual information (PMI) was computed to evaluate the likelihood of a drug-ADR relation. The authors analyzed several case studies of drugs to show that some ADRs were reported prior to the FDA communication. One limitation of this work is the amount of annotated data; the test set contained less than 500 samples. See [39] for a comprehensive review of ADR extraction from social media data with NLP-based approaches.

Supervised machine learning techniques have been successfully applied to detect ADRs. Bian et al. [31] utilized an SVM classifier to identify tweets describing ADRs. Yom-Tov and Gabrilovich [22] analyzed web search query logs to extract information related to drugs and adverse reactions. ADR extraction has been regarded in many studies as a sequence labeling problem using conditional random fields (CRF). CRFs with a rich set of contextual, lexicon-based, grammatical, and semantic features were used in [6, 9, 33]. In [6], the semantic features were based on word clusters using *k*-means clustering on pretrained word embeddings. A set of experiments showed that contextual and semantic features are the most effective to classify ADRs in tweets. We also note a Social Media Mining Shared Task Workshop (organized as part of the Pacific Symposium on Biocomputing 2016) devoted to mining pharmacological and medical information from Twitter, with a competition based on a published dataset [32].

Supervised models tend to work well when trained on fully labeled data. Although there is a large amount of unlabeled data from social media, labeled data are time-consuming to obtain. Gupta et al. [35, 43] used semisupervised learning of patterns to identify drugs, symptoms, and conditions. Lexico-syntactic patterns have been learned with a seed dictionary of terms, and a bootstrapped rule-based method extracted specific entities that were missing from the seed dictionaries. One limitation of this approach is that it does not identify long descriptive phrases. Stanovsky et al. [44] employed an active learning technique to create a bootstrap lexicon of ADRs. The main advantage of this approach is that it can identify entities with a small number of hand-written rules or hand-labeled examples. We mark these works as possibilities for future improvements of this area.

The most relevant studies to the present paper are the works by Metke-Jimenez and Karimi [33], Miftahutdinov et al. [4], and Stanovsky et al. [44]; all of them used the CADEC corpus for training and testing. Metke-Jimenez and Karimi [33] applied dictionary-based methods and CRFs to identify ADRs from the CADEC corpus. They used baseline features, including a bag of words, letter *n*-grams, and word shapes (e.g., if the token composed of uppercase letters). For evaluation, they applied strict and relaxed versions of the evaluation for each matching span. The authors divided the corpus into training and testing sets, using a 70/30 split. CRF outperformed knowledge-based methods on the sentence level and achieved strict and relaxed *F*_1_-measures of 60.2% and 84.9%, respectively. Miftahutdinov et al. [4] applied CRF with a rich set of features to extract all disease-related entities including drug indications, ADRs, and patient history. For CRF features, they used hand-crafted features including contextual features, dictionaries, and cluster-based and distributed word representations. CRF outperformed bidirectional 2-layer and 3-layer RNNs on review level based on 5-fold cross evaluation and achieved *F*_1_-measures of 69.1% and 79.4% on recognition of disease-related expressions in the exact and partial matching exercises, respectively. They used word embeddings trained on social media and on scientific literature separately. Stanovsky et al. [44] employed RNN and word embeddings trained on a Blekko medical corpus in conjunction with embeddings trained on DBpedia. If a word was a lexical match with one of the DBpedia entities, then the DBpedia embedding was used as the input of RNN. Otherwise, Blekko embedding was used. The authors used a 75/25 split and computed evaluation metrics for every word in a sentence instead of extracted spans of ADRs. The knowledge-infused RNN achieved *F*_1_-measures of 93% in the evaluation of each word. The authors did not evaluate RNN in comparison with CRF and LSTM in comparison with GRU. We also note that those papers did not analyze the number of epochs for training RNNs and did not apply the joint model of RNN and CRF.

Our work differs from the mentioned works in several important aspects.

1. We experiment with a joint model of CRF and RNN as well as both models separately.
2. In addition, we employ CNN to extract character-level features instead of engineering of hand-crafted features.
3. We use word embeddings trained on social media and on scientific literature.
4. We present quantitative analysis as well as qualitative analysis of extracted ADRs to demonstrate variation in ADRs across different patient groups.

## 3. Model

This section illustrates our supervised model combining recurrent neural network (RNN) and conditional random fields (CRF) to the extraction of ADRs. We formulate the disease-related entity extraction as a sequence labeling problem. In the following subsections, we describe the architecture of the model.

### 3.1. Recurrent Neural Networks

RNNs are naturally used for sequence learning, where both input and output are word and label sequences. RNN has recurrent hidden states, which aim to simulate memory, that is, the activation of a hidden state at every time step depends on the previous hidden state [45]. The recurrent unit computes a weighted sum of the input signal.

Training RNNs to capture long-term dependencies is difficult due to the effect of vanishing gradients [46], so the most widely used modification of RNN units is the long short-term memory (LSTM) [47] that provides the “constant error carousel” and does not preclude free gradient flow. The most common LSTM architecture contains three gates: input gate, forget gate, and output gate, together with a recurrent cell. LSTM cells are usually organized in a chain, with outputs of previous LSTMs connected to the inputs of subsequent LSTMs. A recent simplification of the LSTM architecture is given by gated recurrent units (GRU) introduced by Cho et al. [48]. GRU is very similar to an LSTM cell; GRU has a single “update gate” instead of separate forget and input gates, does not distinguish cell state and hidden state, and always exposes the entire hidden state, without a special gate for it.

An important modification of the basic RNN architecture is bidirectional RNNs, where the past and the future context is available on every time step [49]. Bidirectional LSTMs, developed by Graves and Schmidhuber [50, 51], contain two chains of LSTM cells flowing in both forward and backward direction, and the final representation is either a linear combination or simply concatenation of their states.

### 3.2. Conditional Random Fields

CRF [52] is one of the state-of-the-art methods that takes a sequence of tokens as input, estimates the probabilities of labels (from a predefined set), and returns the best scoring label sequence. We denote by *x*_1_,…, *x*_*n*_, *x*_*i*_ ∈ *R*^*m*^ corresponding to the input sequence and by *Y* to the labels. The CRF is defined by a graph whose vertices are indexes of *Y* and edge weights correspond to the effects that *X* and *Y* have on each other, given that the Markov property holds. A linear-chain CRF is a CRF with a simple chain as the graph, where each edge has the form of (*j* − 1, *j*).

As shown in [52], the conditional probability of a label sequence is computed as follows:

(1) $$\begin{array}{c} p_{\lambda ,\mu }\left(y\;|\;x\right)=\frac{1}{Z\left(x\right)}\cdot \overset{n}{\underset{t=1}{\prod }}\text{exp}\left.\left(\;\lambda_{y_{t-1}y_{t}}+\langle \mu_{y_{t}},x_{t}\rangle \right.\right), \end{array}$$

where *Z*(*x*) is the normalization, *x* is the feature vector, *μ* is the matrix of size |*Y*| × *m*, *λ* is the matrix of |*Y*| × |*Y*|, and *μ*_*y*_*t*__ is the *y*_*t*_ row in the matrix *μ*. In the equation, the augend represents the score of a transition from the tag *y*_*t*−1_ to the tag *y*_*t*_. In our case, the addend represents the score of the tag *y*_*t*_ of the *t*th word. We define the addend to be the matrix of scores output by the recurrent network. Maximum likelihood learning involves maximizing

(2) $$\begin{array}{c} y=\arg\underset{y\in Y}{\max}\frac{1}{Z\left(x\right)}\cdot \overset{n}{\underset{t=1}{\prod }}\text{exp }\left(\lambda_{y_{t}y_{t-1}}+\langle \mu_{y_{t}},x_{t}\rangle \right). \end{array}$$

We use an implementation of the linear-chain CRF that minimizes the loss function and trains the weights for computing the global tag sequence scores. During testing, the model applies the Viterbi algorithm to predict the best scoring tag sequence.

### 3.3. Joint Model

**Figure 1:**
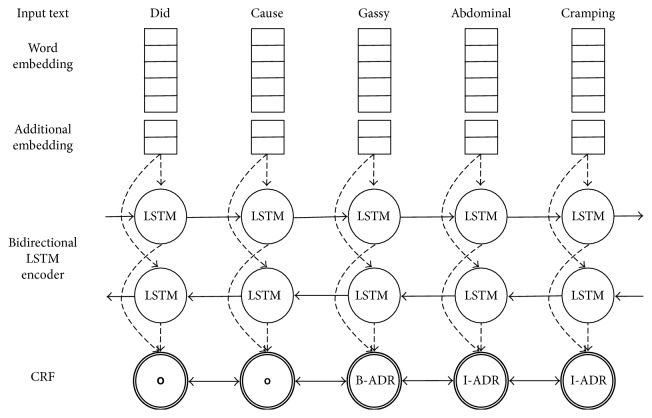
The main architecture of our model. Word embeddings are given as input to the bidirectional LSTM network. Dashed arrows represent the input and output vectors of the network with dropout. The labels follow the BIO (Beginning Inside Outside) tagging scheme.

The main idea of our proposed model is to combine CRF with a neural network, using nonlinear potentials modeled by a neural network instead of linear potential functions based on sparse features.  Figure 1 illustrates the proposed architecture for ADR extraction.

First, word embeddings are fed into the bidirectional RNN (e.g., LSTM). Circles represent LSTM cells. The network returns a representation of the forward and backward context for each word. Then, these output vectors go through a dropout layer for regularization [53]. The result feeds into a dense layer with linear activation, whose output size equals the number of tags. The difference with standard RNN architecture is that we do not use the softmax output from this layer directly but rather utilize the output of the dense layer for an additional CRF layer to jointly decode the sequence of context tags.

Another important part of the model is the extra vector marked as “additional embedding” on Figure 1. In the experiments shown below, we augmented the basic word embeddings with an additional vector trained with a character-level CNN [16], simply concatenating the two vectors as input for the bidirectional LSTM; we will see that this additional model also improves the final results.

## 4. Experiments and Discussion

### 4.1. Quality Metrics and Datasets

In this section, we evaluate our model and compare it with baseline approaches. Since the boundaries of expressions are hard to define even for human annotators [54], we follow [55, 56] and conduct the experimental evaluation as follows:

1. Exact matching following CoNLL evaluation [57]
2. Partial matching as described in [56].

We computed several model accuracy metrics such as macroaveraged precision (*P*), recall (*R*), and *F*_1_-measure (*F*) as follows:

(3) $$\begin{array}{c} P=\frac{TP}{TP+FP},\\ R=\frac{TP}{TP+FN},\\ F=\frac{2\;\cdot \;P\;\cdot \;R}{P+R}, \end{array}$$

where TP is the number of correctly predicted annotations and FP and FN are the numbers of false positives and false negatives, respectively. Following [56], we used the following formulas for partial matching:

(4) $$\begin{array}{c} P=\frac{\left|t\cap t_{s}\right|}{\;|\;t_{s}\;|\;},\\ R=\frac{\left|t\cap t_{s}\right|}{\;|\;t\;|\;}, \end{array}$$

where *t*_*s*_ is an extracted term which intersects with the term *t*, *t*_*s*_∩*t* is the intersection between *t* and *t*_*s*_, and ∣*t*∣ is the length of this term in tokens. For partial matching, we calculated metrics for every sentence and averaged the resulting values.

We used the Keras library (https://keras.io/) to implement neural networks and the BIO (Beginning Inside Outside) tagging scheme on the sentence level. The batch size was 128; we used the Adam optimizer with default parameters [58]. We evaluated our network using the high-quality annotations from the CADEC corpus. Similar to [33], we excluded overlaps between spans of ADRs in the CADEC corpus, selecting the longest continuous span and combining these ADRs into a single annotation.

The corpus was split into two different datasets, leaving 70% for training (with a total of 875 reviews, 5264 sentences, and 3933 ADRs) and 30% (375 reviews, 2356 sentences, and 1837 ADRs) for testing.

### 4.2. Experimental Results

We evaluate our model by comparing with the following methods:

1. CRF with the following baseline features: each word itself with a part-of-speech tag, the suffixes and prefixes to 6 characters in length, and a window of two words in both directions (backward and forward) from the current word
2. Feature-rich CRF-based approach with parameters as proposed in [4]; this method utilizes the following features: baseline contextual features, dictionaries, and cluster-based and distributed word representation. The authors used the following dictionaries: the Unified Medical Language System (UMLS), ADR lexicons, and a dictionary of multiword expressions such as “feel tired,” and “feel sleepy.” The Brown hierarchical algorithm was used for cluster-based word representations (vector size of 150). The authors trained Continuous Bag of Words model on a corpus of health-related reviews with the following parameters: vector size of 200, the length of local context of 10, negative sampling of 5, and vocabulary cutoff of 10. We used publicly available implementation of this feature-rich approach (https://github.com/dartrevan/ChemTextMining/)
3. Deep bidirectional RNNs with a softmax layer, in particular, LSTM and GRU, where the combination of the network's outputs is fed into a fully connected layer with softmax activation.

We used a maximum of 100 epochs to train each network. For fair comparison, all networks used the same word embeddings trained on 2.5 million of health-related reviews [4]. We found 97% of words in the vocabulary, and for 3% of words, the representations were uniformly sampled from the range of embedding weights [59]. The results of different methods are shown in Table 1.

Table 1 shows that the proposed model consistently outperforms other approaches in terms of both precision and *F*-measure, while staying roughly on par with the best recurrent models in terms of recall. Therefore, we conclude that a combination of RNN and CRF indeed leads to quality improvements for ADR extraction from free-text reviews. The second conclusion is that concatenating input word embeddings with an extra embedding vector based on a character-level CNN also significantly improves the results. Another interesting conclusion from Table 1 is that GRU-based recurrent architectures consistently outperform LSTM-based architectures in the exact matching exercise. Finally, another interesting conclusion is that *F*_1_-scores of 3-layer GRU + CNN + CRF increased from 70.65% to 79.78% in the partial matching as compared to the exact exercise due to boundary problems. Qualitative analysis of results indicates errors associated with boundaries of entities due to the presence of negations (e.g., “I have *no pain*”), conjunctions, verbs, adjectives, or adverbs (e.g., “*lowered total cholesterol* dramatically”).

**Figure 2:**
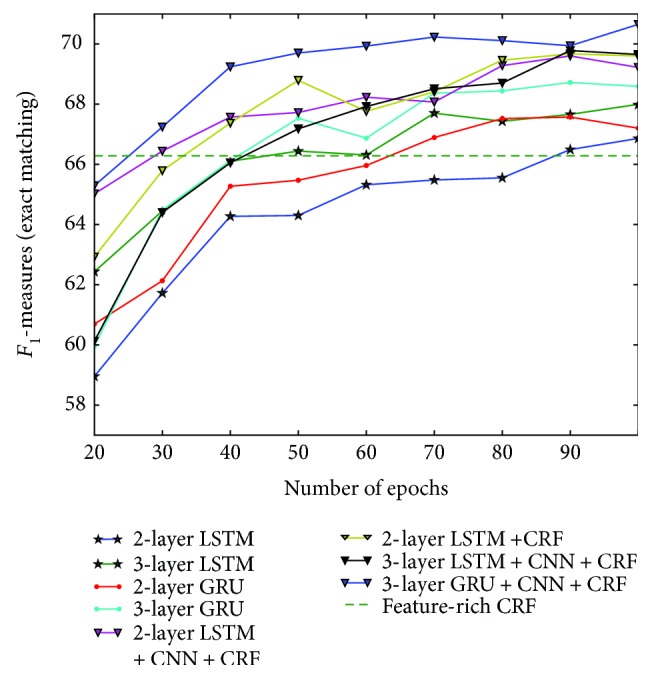
Performance on the testing data set or different number of training epochs.

We initially set the number of epochs for training models to be 100 and explored the quality metrics for the number of training epochs ranging from 20 to 100.  Figure 2 presents the results. It shows that training of deep LSTM and GRU can be effectively achieved at around 60–80 epochs before the performance becomes stable. The joint model of 2-layer LSTM + CNN + CRF and 3-layer GRU + CNN + CRF outperformed CRF starting at 30–40 epochs, and from here on, performance improved slowly.

### 4.3. Qualitative Analysis of Extracted ADR Mentions

Adverse drug reactions can differ significantly depending on the patient. To investigate the difference between adverse effects for various drugs, we collected reviews from a health information service webmd.com. Each review contains the following fields:

1. *Brand name* of a drug used to treat this disease
2. *Condition/reason* for taking treatment
3. *The free-text review* given for the effects caused by the use of the drug
4. *Demographic information* about the author of this review (age and gender).

We also note that such demographic information is not commonly provided in discussion groups and websites. In a recent study [60], several approaches to automated mining of demographic information from texts about drugs were evaluated including neural networks, supervised machine learning, and topic modeling.

We selected reviews about the following health conditions for analysis:

1. 4,563 reviews about depressive disorder (drugs: Cymbalta, Lexapro, Xanax, Zoloft, or Prozac)
2. 5,422 reviews about high blood pressure (drugs: lisinopril, atenolol, Bystolic, Diovan, and hydrochlorothiazide)
3. 10,914 reviews about fibromyalgia (drugs: Cymbalta, Lyrica, tramadol, Prozac, amitriptyline, Savella, Paxil CR, Ultram, Paxil, cyclobenzaprine).

In order to detect ADRs related to a particular demographic group, we extracted all ADRs that appeared in reviews more than four times and then excluded ADRs if the exact match appears in reviews of authors with a different demographic tag (e.g., “male”/”female” or “age 19–34” over other ages). Tables 2, 3, and 4 present the results. The tables indicate that key adverse reactions change with age or gender, reflecting quite natural progressions that match well with medical and commonsense intuition. Hence, our method can also be used to mine qualitative information from a dataset of medical reviews, perhaps uncovering new ADRs in a certain user group.

## 5. Conclusion

In this work, we have proposed a novel approach to extracting adverse drug reactions from user reviews: a combination of a bidirectional LSTM-based recurrent neural network and a CRF that operates on the scores extracted by this neural network. We have evaluated our approach against state-of-the art neural models on a representative ADR extraction dataset and have found that the results have improved significantly. Moreover, further improvements were obtained by extending input embeddings with a character-level model. Thus, our final model successfully combines three different approaches to statistical modeling in NLP. In further work, we plan to experiment with other neural models in similar general architectures and further improve the state of the art in ADR extraction from free-text reviews.

## Figures and Tables

**Table 1:** Results of the proposed models and baseline methods.

Method	Exact			Partial		
	*P*	*R*	*F*	*P*	*R*	*F*
Baseline CRF	0.6254	0.5972	0.6110	0.8145	0.7539	0.7521
Feature-rich CRF	0.6726	0.6532	0.6628	**0.8303**	0.7646	0.7622
1-layer LSTM	0.5798	0.6587	0.6167	0.8121	0.8065	0.7809
2-layer LSTM	0.6362	0.7044	0.6686	0.8090	0.8495	0.8005
3-layer LSTM	0.6588	0.7022	0.6798	0.8247	0.8323	0.7997
4-layer LSTM	0.6689	0.7093	0.6885	0.8255	0.8280	0.8000
1-layer GRU	0.5862	0.6772	0.6284	0.7995	0.8368	0.7900
2-layer GRU	0.6384	0.7093	0.6720	0.8165	0.8338	0.8002
3-layer GRU	0.6675	0.7191	0.6923	0.8151	0.8373	0.8009
4-layer GRU	0.6565	**0.7262**	0.6896	0.8006	**0.8665**	0.8033
2-layer LSTM + CRF	0.6947	0.6973	0.6960	0.8191	0.8161	0.7872
2-layer LSTM + CNN + CRF	0.6809	0.7039	0.6922	0.8083	0.8488	0.7978
3-layer LSTM + CNN + CRF	0.6868	0.7066	0.6965	0.8270	0.8488	**0.8115**
3-layer GRU + CNN + CRF	**0.7048**	0.7082	**0.7065**	0.8219	0.8311	0.7978

**Table 2:** ADRs extracted from reviews for the drugs that treat depression.

Group	Adverse drug reactions
All authors	Anxiety, depression, panic attacks, depressed, pain, weight gain, nausea, headaches, dizziness, insomnia, dizzy, mood swings, tired, dry mouth, sweating
Gender group “female”	Rash, gained weight, could not sleep, heartburn, severe nausea, lost weight, restless, very irritable, heart racing, disconnected, stiffness, upset, severe migraines, cramping, neck pain, twitching, fever, skin problems
Gender group “male”	Erectile dysfunction, pins and needles, burning sensations, loose bowels, urination, uneasiness, trouble with dizziness, severe drowsiness, night sweat, chest pressure, blisters, clammy hands
Age group “19–34”	Couldn't sleep, anger issues, loss of sex drive, cramps, unmotivated, jaw pain, frequent headaches, fever, stomach pains, crying for no reason, severe dizziness, intrusive thoughts
Age group “45–64”	Nervous breakdown, aches and pains, swelling, muscle aches, delayed ejaculation, profuse sweating, indigestion, ringing in my ears, spasms, trouble urinating, palpitations

**Table 3:** ADRs extracted from reviews for the drugs that treat high blood pressure.

Group	Adverse drug reactions
All authors	Cough, coughing, dizziness, dizzy, headaches, dry cough, fatigue, tired, headache, weight gain, hair loss, nausea, anxiety, shortness of breath, tiredness, diarrhea, chest pain, depression, joint pain, rash, swelling, very tired, light headed, blurred vision
Gender group “female”	Heart palpitations, hives, gagging, hot flashes, extremely tired, nightmares, chronic cough, cold hands and feet, panic attacks, exhausted, weight loss, blurry vision, heartburn, sleepy, persistent cough, severe headaches, stomach pain, numbness
Age group “45–64”	Bloating, muscle aches, persistent cough, indigestion, stomach pain, post nasal drip, sick, lack of sleep, ringing in my ears, stomach pains, foot cramps, tightness in chest, falling out, severe coughing, faint, nagging cough, no energy
Age group “25–44”	Short-term memory loss, slight weight gain, fast heartbeat, lost sex drive, cramp, unusual tiredness, bad dreams, numbness in my toes, pain in my side, dazed feeling, intense salt cravings, lip to swell, chronic headaches, throat and neck swelled

**Table 4:** ADRs extracted from reviews for the drugs that treat fibromyalgia.

Group	Adverse drug reactions
All authors	Pain, depression, anxiety, weight gain, nausea, headaches, depressed, dizziness, dizzy, panic attacks, nerve pain, insomnia, dry mouth, constipation, sweating, tired, headache, fatigue, back pain, mood swings, hot flashes, nightmares, suicidal thoughts, severe pain, blurred vision, muscle pain, vomiting, chronic pain, suicidal, neuropathic pain, drowsiness, trouble sleeping, sex drive, diarrhea, seizures, crying, anxious, nauseous, numbness, swelling, leg pain, night sweats, vertigo, tremors, joint pain, itching, burning, panic attack, sleepiness, drowsy
Gender group “female”	Severe migraines, water retention, severe panic attacks, suicidal ideation, exhaustion, stiff, inability to sleep, rapid heartbeat, crazy dreams, sweaty, nervous breakdown, extreme sweating, fogginess, flushing, major weight gain, increased my appetite
Gender group “male”	Blisters, premature ejaculation, foot neuropathy, burning discomfort, can barely walk, pain in my toes, anger problems, loss of libido, pancreatitis, pain in lower back, hiccups, shock sensations, couldn't walk, can't walk, panic problems, “shock” sensations, hangover, short-term memory, severe trouble urinating
